# Supplementary material for: Splice-Junction-Based Mapping of Alternative Isoforms in the Human Proteome
Source: Cell Rep. Author manuscript; Available in PMC 2020 Jan 15. (PMC6961840; doi:10.1016/j.celrep.2019.11.026)
Supplement: 2 [file NIHMS1546469-supplement-2.zip › DF1/PXD010154/Lung/Lung_46_CASC1_ILGVILR.pdf]

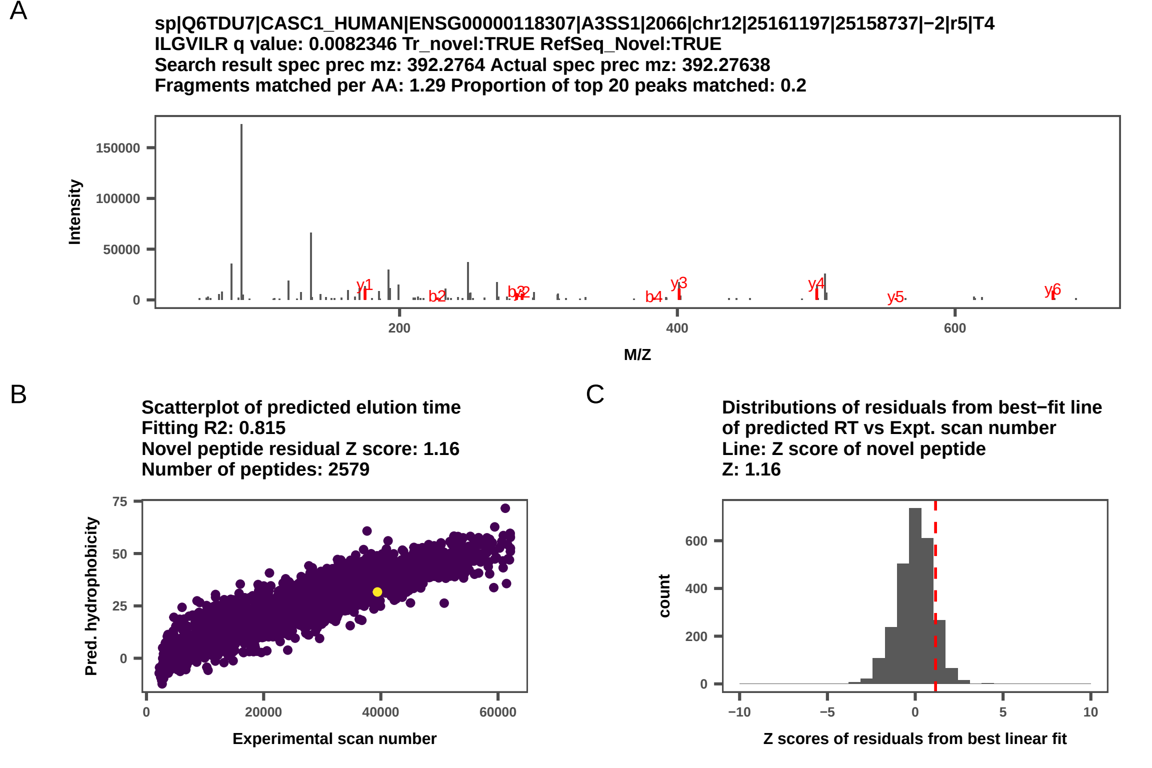
A
sp|Q6TDU7|CASC1_HUMAN|ENSG00000118307|A3SS1|2066|chr12|25161197|25158737|−2|r5|T4
ILGVILR q value: 0.0082346 Tr_novel:TRUE RefSeq_Novel:TRUE
Search result spec prec mz: 392.2764 Actual spec prec mz: 392.27638
Fragments matched per AA: 1.29 Proportion of top 20 peaks matched: 0.2
Intensity
150000
100000
50000
0
y1
b2
b3
y2
b4
y3
y4
y5
y6
200
400
600
M/Z
B
Scatterplot of predicted elution time
Fitting R2: 0.815
Novel peptide residual Z score: 1.16
Number of peptides: 2579
Pred. hydrophobicity
75
50
25
0
0
20000
40000
60000
Experimental scan number
C
Distributions of residuals from best−fit line
of predicted RT vs Expt. scan number
Line: Z score of novel peptide
Z: 1.16
count
600
400
200
0
−10
−5
0
5
10
Z scores of residuals from best linear fit
